# Supplementary material for: The role of innate immune responses against two strains of PEDV (S INDEL and non-S INDEL) in newborn and weaned piglets inoculated by combined orogastric and intranasal routes
Source: Front Immunol. 2025 Jun 16;16:1584785. doi: 10.3389/fimmu.2025.1584785 (PMC12206634; doi:10.3389/fimmu.2025.1584785)
Supplement: Supplementary file 1 [file Table1.docx]

Supplementary Material

# Supplementary Data

## Supplementary Figure 1

**Supplementary Figure 1.** Comparative IFN I and III intestinal gene expression in suckling (5d) and weaned (5w) piglets inoculated with PEDV CALAF and PEDV USA strains.
